# A case study on using quantile regression in psychiatry research

**DOI:** 10.3389/fpsyt.2025.1632001

**Published:** 2026-01-05

**Authors:** Ravi G. Shankar, Thennarasu Kandavel, Himani Kashyap, Y. C. Janardhan Reddy

**Affiliations:** 1 Department of Biostatistics, National Institute of Mental Health and Neuro Sciences, Bengaluru, Karnataka, India; 2 Department of Clinical Psychology, National Institute of Mental Health and Neuro Sciences, Bengaluru, Karnataka, India; 3 Department of Psychiatry, National Institute of Mental Health and Neuro Sciences, Bengaluru, Karnataka, India

**Keywords:** quantile regression, linear regression, neuropsychological tests, obsessive-compulsive disorder, non-normal distribution

## Abstract

Commonly used linear regression focuses only on the effect on the mean value of the dependent variable and may not be useful in situations where relationships across the distribution are of interest. This study aimed to appraise the utility of Quantile Regression (QR), a technique that can model any quantile value of the dependent variable. The primary aim of this study is to provide an overview of the QR method and its practical applications in psychiatric research. We demonstrated this with an exploratory analysis of the data on neuropsychological test performance among 119 subjects with obsessive-compulsive disorder (OCD). The varying effects of age, education, sex, antipsychotic use, and symptom severity between extreme quantiles were highlighted using simple and multiple QR models. While linear regression is easy to employ and interpret, QR is not only on par in performance but also more flexible in identifying a set of factors that may differ depending on the quantile of interest. QR analysis is a potent tool in applications where the effect of the independent variable varies depending on the values of the outcome variable. The results of this exploratory study suggest that the QR approach could potentially help explore inconsistent findings, generate future hypotheses, and/or provide possible interpretive frameworks for inconsistencies observed in neuropsychological research in OCD. As the QR offers a complete distributional analysis, it is valuable in providing new insights, especially in situations where the usual regression assumptions are violated or when interested the in extreme values of the outcome of interest.

## 1 Introduction

Linear regression, the most widely used method among a broad family of regression methods, models a continuous dependent variable (DV) as a function of categorical or continuous independent variables (IV). Ordinary least squares (OLS), a technique for estimating regression coefficients, is the most appealing because of its simplicity in interpretation, practical utility, and well-developed theoretical framework (1). However, the validity and reliability of the results with OLS regression are largely dependent on assumptions such as normality and homogeneity (equal variances) of error terms. The estimates can be misleading, and the statistical conclusions drawn may be invalid in OLS regression if these assumptions are not met (2, 3). The use of the OLS method without testing assumptions and despite violations of assumptions is common (4) as it is commonly available to employ in popular statistical software packages.

In general, conditional mean regression models, such as linear regression, reveal the effect of IVs only on the mean (representing the central location of a distribution) change in the DV but not on other values/locations of its distribution. Sometimes, it is important to see the effects of IV on non-central locations like quartiles or a certain specific extreme part of the distribution, such as the 5th or 95th percentile. Because conditional mean models fail to model quantiles, they provide an incomplete picture of the possibly different magnitude of the effect of an IV across the distribution of the DV. They invariably assume the same effect throughout the distribution; however, it is not necessary for a variable to have an equal effect on all parts, as it may depend on the values of the response variable. While assuming so may lead to missing out on important differential effects, treating them to have variable effects may reveal a different set of covariates for different parts of the distribution (5, 6).

Alternatives to traditional linear regression continue to intrigue applied statisticians and methodological researchers because of the limitations of the classical linear regression technique (7). Based on Mosteller and Tukey’s (8) idea of extending conditional mean models, Koenker and Bassett (9) proposed a concept of Quantile Regression (QR) to fit regression curves to specified parts of the distribution other than mean. QR is a natural extension of conditional mean models to other locational parameters, and it aims to estimate and conduct inference about any conditional quantile values in place of the mean (9). A special case of QR is called median regression, which is most appropriate in the case of a skewed distribution. QR coefficients describe how a specified quantile of the response changes per unit change in the predictor, as opposed to the mean change in the traditional OLS regression.

QR effectively deals with the problem of expressing the relationship with only one regression curve, as in OLS. This is especially useful when the DV is heavy-tailed/non-normally distributed, if the effects of covariates differ at different tails of the distribution, and when the extreme values (low and high) are important and/or are non-ignorable. For example, the data on neuropsychological performance of individuals often violate assumptions as they show higher variability, and it is difficult to apply many of the standard statistical procedures in such situations (10). It has also been reported that the results involving such variables are inconsistent across different studies (11). For instance, neuropsychological research on obsessive–compulsive disorder (OCD) is marked by inconsistent and heterogeneous findings despite decades of study (12). Given the emphasis placed on neuropsychological functions in clinical and functional recovery in psychiatric disorders (13), statistical methods may be useful in exploring potential sources of inconsistencies across studies. Neuropsychological dysfunction in OCD is significant and may persist despite clinical remission (12, 14) and predict treatment outcomes (15, 16). Neuropsychological findings are linked to the cortico-striatal-thalamo-cortical circuit, which is posited to underpin the clinical symptoms of OCD (17).

Some studies (18–20) have emphasized the utility of QR in behavioral sciences using real-life and simulated datasets. However, older methods, such as OLS regression, predominate the literature despite their limited applicability in specific situations. The main objective of this study is to provide an overview of the QR method and its practical applications using open-source R software. The secondary aim is to demonstrate the utility of this method with a strictly exploratory analysis of the data from neuropsychological test performances among individuals with OCD. This manuscript is primarily intended for methodologists and methodologically oriented clinical researchers; therefore, the clinical analyses are presented strictly as an illustrative case study.

## 2 Methodology

### 2.1 Estimation in QR

The unknown parameters $\beta_{i}$ in linear regression models are usually estimated using methods such as OLS, based on minimizing the sums of squared residuals. The idea of minimizing squared residuals can be extended to minimize absolute residuals to yield a line with minimum absolute error. QR methods offer an equally convenient mechanism for estimating models for the full range of conditional quantiles. The minimization of QR is reformulated as a linear programming problem and solved using algorithms. Several existing algorithms have been efficiently implemented using new-generation computers and software (21). For any quantile τ ∈ (0, 1) defined on the distribution of the DV, the quantity β(τ) is called the *τ*th regression quantile.

### 2.2 Statistical inferences in QR

The methods for computing confidence intervals and statistical inferences can be classified as direct methods based on the asymptotic normality of the estimated regression quantiles, the rank-score method based on the inversion of the rank-score test, and the resampling methods, which use the bootstrap technique and other procedures. The widely used goodness of fit of a linear regression using R^2^ was extended to QR models by Koenker and Machado (22) via a pseudoR^2^ measure keeping in view of the difference in the estimation technique. Similar to the usual R^2^, it ranges between 0 and 1, with a higher value indicating a better model fit, but it only pertains to the particular quantile; hence, each fitted QR model will have a separate pseudo R^2^ value. The same measure can be used to test the significant difference between nested models, which will yield an estimate of the relative pseudoR^2^. In addition, information criteria such as Akaike (AIC) and Bayes (BIC) can be used to compare the overall model fit. The Likelihood Ratio Test (LRT) can be used to measure and verify the inclusion/exclusion of one or a set of variables under the QR framework, unlike in OLS, where different thresholds, such as p-values, are used to develop the best model.

Although the QR method addresses the issue of varying covariate effects across quantiles by estimating different regression lines, it is also important to test the equivalence of a pair of QR lines across quantiles corresponding to the same covariate. This test can determine whether all of the conditional quantile functions have the same intercept/slope parameter. An Analysis of Variance (ANOVA) type of scenarios is derived to test hypotheses about the equivalence of estimates across quantiles using asymptotic results (21). There are also newer methods that look beyond traditional p-value based interpretations, such as quantile loss approaches, which measure the model performance by evaluating calibration, discrimination, and overall performance of quantile predictions from the fitted model (23).

### 2.3 Data and study variables

Real-life data from a previously published cross-sectional study were used to demonstrate the utility of the QR approach. The participants were recruited from the OCD Clinic, Behavioral Medicine Unit, and Department of Psychiatry, National Institute of Mental Health and Neuro Sciences, Bengaluru, India, between January 2008 and 2010 (24). The subjects were diagnosed with OCD according to the DSM-IV criteria based on a detailed clinical interview using a semi-structured *pro forma* and the Mini International Neuropsychiatric Interview Plus (MINI Plus version 5.0). For this exploratory analysis, selected variables from a comprehensive battery of neuropsychological tests, such as attention, executive functions, memory, and intelligence, were used. Participants with complete data on all sociodemographic, clinical variables, and test scores (n = 119) were included in the current study. The exclusion and inclusion criteria for the participants and variable operationalization/definitions for the data utilized in this study are described in the supplemental section and elsewhere (24). Although the data were collected between 2008 and 2010, they were used here to address the primary objective of demonstrating the methodological application of Quantile Regression rather than to derive time-sensitive clinical conclusions.

### 2.4 Comparison of regression approaches

Linear regression is an obvious method of choice for identifying the factors that affect performance. Because of the skewed distribution of the DV and the importance of assessing the extreme parts of the DV, both conditional mean regressions using OLS and QR models at every 10th quantile ranging from 0.1 to 0.9 along with the 5th, 25th, 75th, and 95th quantiles were fitted. Bivariate and multivariate QR models were fitted with each neuropsychological performance as the dependent variable. Bootstrap-based confidence intervals were calculated for the coefficients and tested for their statistical significance. The models also included current age and sex as they are common influencing factors for neuropsychological scores (10), along with other variables such as years of education, age of onset of OCD, symptom severity scores, and drug use. Conditional normality and homogeneity of variances for the OLS model were tested using the Shapiro–Wilk and Breush–Pagan tests, respectively. An ANOVA-based approach was used to test the equality of the QR coefficient fit for two different quantiles. Model fits were assessed using measures such as AIC and pseudo-R^2^.

All the estimations and inference procedures stated above for the empirical analysis in this study were carried out using open-source software R (25), where most QR-related functions are available in the *quantreg* package (26).

## 3 Results

The patients’ profiles in terms of sociodemographic and selected clinical characteristics are displayed in **Supplementary Table 1**. The scores on the selected tests showed a high range of variability and skewed distributions, as shown in **Supplementary Figure 1** and **Supplementary Table 2**. Many subjects scored very high/low, deviating from the average score, and a formal Shapiro–Wilk’s test for these indicated a non-normal distribution in most cases.

**Figure 1:**
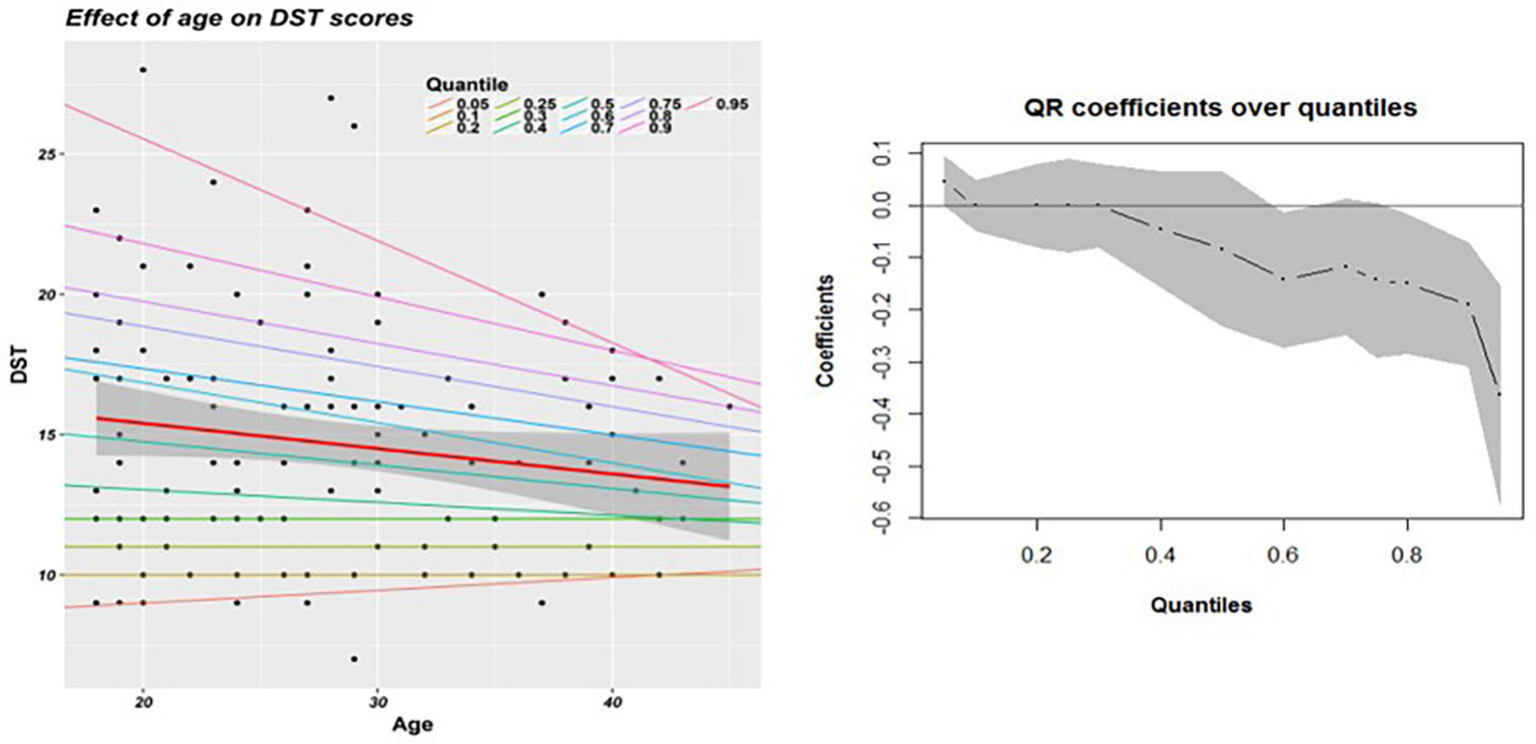
**(A)** Simple regression models with effect of age on DST scores. **(B)** Change in QR coefficients across various quantiles.

The results of the model with only age as an IV in modeling digit span test (DST) scores are presented in **Figures 1A, B**, whereas **Table 1** shows the results in numbers along with results of other variables. The variable DST was selected to explain the results of the QR model, and a similar explanation holds for the other variables. The scatterplot of age with DST scores shows the red line indicating the OLS regression fit with its estimated confidence intervals superimposed with QR lines for each selected quantile. **Figure 1B** shows the estimates and confidence intervals of the regression coefficients across quantiles, which helps examine the trend via visualization. The non-significant regression coefficient using OLS (b = −0.09, p = 0.09) regression indicates a marginal decrease in DST scores for a yearly increase in age, but the test of residuals indicates a violation of the assumption of normality (p = 0.002). In contrast, the coefficients from the QR model change from a smaller, non-significant but positive value at the 5th quantile (b = 0.05, p = 0.11) to a higher, significant negative value at the 95th (b = −0.36, p =<0.001). The effect of age on DST is averaged out in OLS and QR, revealing that an increase in age leads to a greater decline in DST scores along the 95th quantile of DST values than along the 5th quantile.

**Table 1:** Simple QR model—comparison of OLS and QR across selected quantiles for the model with age as independent variable.

Dependent variable	OLS regression	Regression coefficient (p-value) (95% confidence interval)				
		Quantile values				
		5th (lower extreme)	25th (1st quartile)	50th (Median)	75th (3rd quartile)	95^th^ (upper extreme)
DST	−0.09 (0.090) (−0.19, 0.01)	0.05 (0.110) (**−**0.02, 0.11)	0.00 (1.000) (-0.11, 0.11)	**−**0.0 (0.350) (**−**0.25, 0.09)	**−**0.14 (0.110) (**−**0.32, 0.04)	**-0.36 (<0.001)** **(-0.62, -0.11)**
CTT2	**1.76 (0.010)** **(0.46, 3.05)**	0.71 (0.330) (**−**0.65, 2.06)	**1.17 (0.040)** **(0.14, 2.20)**	**1.86 (0.010)** **(0.45, 3.27)**	**2.57 (0.040)** **(0.18, 4.97)**	2.09 (0.210) (-1.06, 5.23)
CFT_IR	**−0.18 (0.020)** **(−0.32, -0.03)**	**−**0.25 (0.280) (**−**0.69, 0.19)	**−**0.17 (0.160) (**−**0.40, 0.06)	**−**0.19 (0.070) (**−**0.38, 0.01)	**−**0.08 (0.610) (**−**0.39, 0.23)	0.00 (1.000) (-0.21, 0.21)
TOH3 moves	**3.28 (0.020)** **(2.58, 4.26)**	0.00 (1.000) (**−**0.02, 0.02)	0.00 (1.000) (**−**0.23, 0.23)	0.00 (1.000) (**−**0.22, 0.22)	0.06 (0.760) (**−**0.34, 0.45)	-0.22 (0.760) (-1.65, 1.20)
WCST_PR	0.39 (0.060) (**−**0.02, 0.79)	0.11 (0.460) (**−**0.18, 0.40)	0.31 (0.260) (**−**0.23, 0.84)	0.35 (0.320) (**−**0.35, 1.05)	0.23 (0.320) **−**0.18, 0.65)	1.05 (0.170) -0.29, 2.40)
WCST_TTCC1	10.22 (0.180) (8.21, 11.45)	0.00 (1.000) (-0.07, 0.07)	0.00 (1.000) (**−**0.04, 0.19)	1.00 (0.860) (**−**0.16, 0.89)	13.00 (0.440) (**−**1.10, 4.70)	0.00 (1.000) (-0.9, 0.9)

OLS, Ordinary Least Squares Regression; DST, Digit Span Test; CTT2, Color Trails Test (CTT); CFT_IR, Complex Figure Test—Immediate Recall; TOH3_moves, Tower of Hanoi Test; WCST, Wisconsin Card Sorting Test.Bolded values in the table indicate significant coefficients at 5% level of significance.

The differential effect of age with respect to other test scores as dependent variables is displayed in **Table 1**. Selected quantiles are displayed for brevity, although 13 different QR models (every 10th quantile along with the 5th, 25th, 75^th^, and 95th) along with OLS were fit for comparison and visualization. **Supplementary Figure 2** summarizes the results of all such QR and OLS models, where most of the OLS models violate the assumption of normality of residuals and homoskedasticity. Variables such as DST, CTT2, and WCST_PR showed an increasing absolute effect of age along the higher quantiles. Age had no effect on WCST_TTCC1 at either extremes, with variations showing only in the middle quantiles. The OLS regression coefficients miss these differential effects and provide an incomplete picture by averaging them out, as can be clearly seen from these results.

Although we can fit as many QR as we may need, the coefficients may not be statistically significant between the two fitted quantiles. To test the equivalence and statistical significance between a pair of QR coefficients corresponding to the same covariate, an ANOVA approach was used, and the results for such tests conducted between the 5th *vs*. 95th and 25th *vs*. 75th quantiles for the model with age as a predictor are presented in **Table 2** (also includes results from multivariable models). Although the tested pairs of coefficients are not significantly different for most of the variables, the significant difference in DST scores for both pairs is highlighted. This further supports the interpretation of the change in the QR coefficient values across quantiles. It is also interesting to note that if a binary predictor variable, such as gender, is used in a simple QR model, a complete distributional comparison is possible, as opposed to a comparison of only the means (analogous to an independent samples t-test) in the case of OLS.

**Table 2:** Testing for equality of slope estimates across selected quantiles for bivariate model and between models with same variables across selected quantiles for multivariable models.

Dependent Variable	Simple model		Multivariable model	
	25th *vs* 75th quantile	5th *vs* 95th quantile	25th *vs* 75th quantile	5th *vs* 95th quantile
DST	0.04	0.01	0.012	<0.001
CTT2	0.19	0.81	0.068	<0.001
CFT-IR	0.55	0.27	0.927	<0.001
TOH-3moves	0.75	–	0.004	0.936
WCST_PR	0.79	0.57	0.152	0.014
WCST_TTCC1	0.38	–	0.643	<0.001

The values in each cell are the p-values from testing the significant differences between the specified quantiles based on ANOVA procedure. A significant p-value indicates differential effect of the independent variable between the specified quantiles. P-values could not be computed in some cases because of data sparsity.

**Figure 2:**
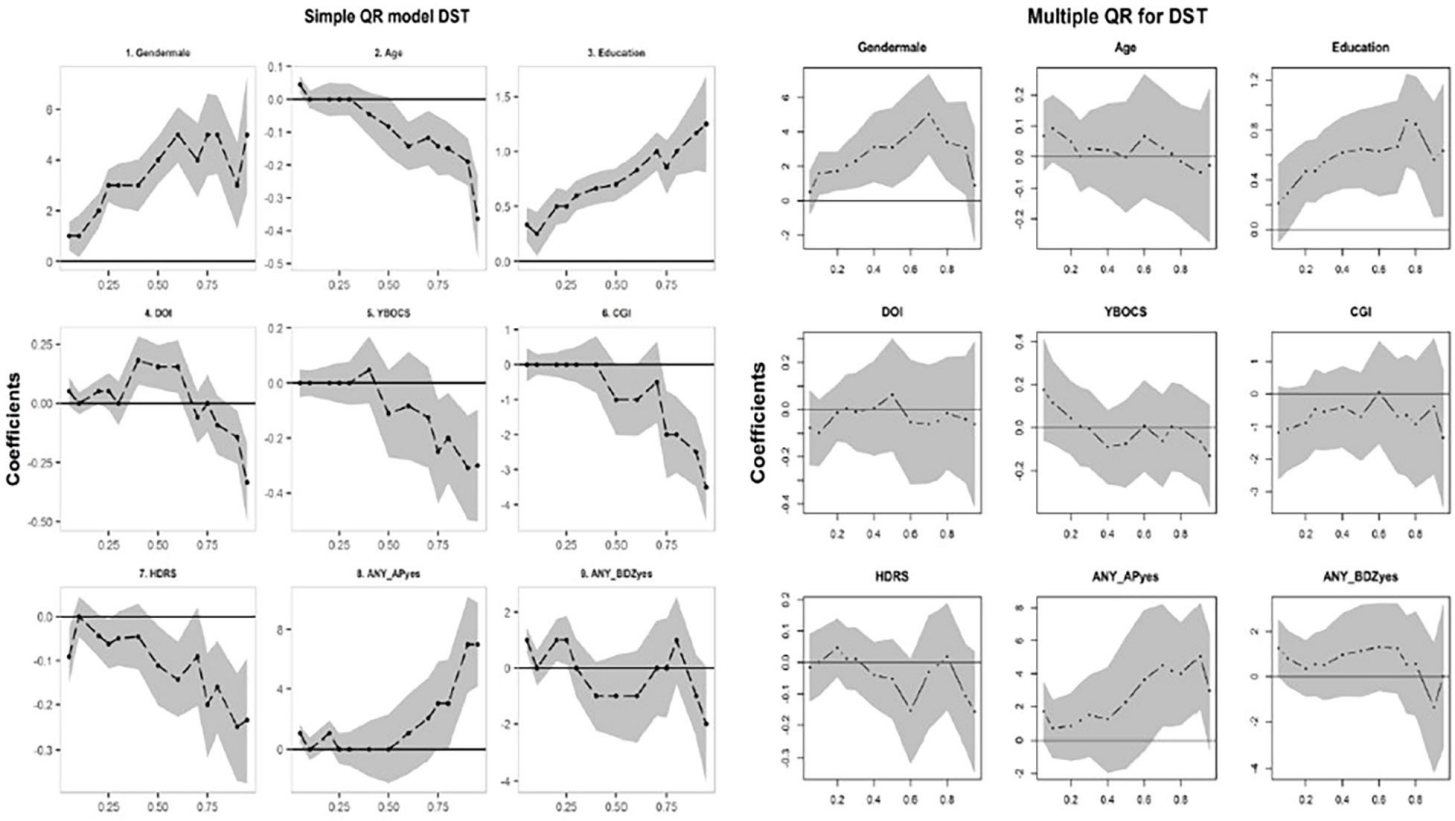
Comparison of change in regression coefficients across quantiles between **(A)** simple and **(B)** multivariable models for DST.

As the next step in the case of other regression model building processes, different sociodemographic and clinical factors that might affect neuropsychological performance, such as sex, age, education, duration of illness, OCD severity (YBOCS), depression rating scores (HDRS), CGI scores, and use of antipsychotics and benzodiazepines, were included in a multivariable model. Comparative graphical presentations of coefficients under separate bivariate and multivariable models for DST scores are shown in **Figure 2**. The right panel shows the coefficients from the adjusted model, and the left panel shows those from the unadjusted model. Adjusted for other variables, the difference in DST scores between sexes increased at higher quantiles, as did the effect of education and antipsychotic intake. The adjusted effects of the YBOCS, HDRS, and CGI were mostly non-significant, which showed a significant effect individually. The effects of education were predominant in both scenarios, indicating a strong association with the scores. Specifically, the positive effects of education are high among higher quantile values. The differential effects of medications were also apparent in the QR analysis. Participants with higher scores were found to have a larger effect with the use of antipsychotic drugs. Furthermore, the supplementary sections (**Supplementary Figure 3**; **Supplementary Table 3**) with the results for models with other test scores show similar trends. The pairwise testing between the two QR models is found to be significantly different between the two extreme quantiles, that is, the 5th *vs* 95^th^, as shown in **Table 2**. Consequently, the set of significant variables in a lower quantile can differ from that in a higher quantile.

The model fit was assessed using pseudo R^2^, and the values for each model are graphically represented in **Supplementary Figure 4**. The aim of this study here was not to establish a superior model but to illustrate how the QR can complement traditional mean-based approaches. Most models showed moderate goodness of fit, indicating the usefulness of IVs in explaining the variation in test scores. DST shows a poor fit at lower quantiles, but the fitness gradually increases for higher quantiles. Relatively better model fitness values were obtained for variables such as CTT1, CTT2, CFT_IR, and WCST_TTCC1 at higher quantiles.

## 4 Discussion

The overall purpose of this study was to employ, explore, and highlight the utilities of the QR method over traditional linear regression in exploring relationships. It is common to use linear regression even when the assumptions are violated (27) because of its ease of use and interpretation, alongside its availability in commonly used software packages. Using QR is advantageous in situations where the assumptions of linear regression are not met, and it is a default choice when the research question itself centers around the effect on the specific percentile of the variable of interest.

It is well known that most variables observed in empirical research fail to follow normality and are skewed in nature, necessitating the use of methods that do not assume normality. This is especially true in the case of social and behavioral sciences (10), where the expected phenotypic variability is high. The QR analysis in the present study showed that it may be useful for better understanding relationships, specifically non-normally distributed outcome variables. The simple QR model showed that the assumption of normality of errors was violated, and the effects of IV varied depending on the quantile being estimated. For example, age may have a significant effect on patients with higher DST scores but not on those with low scores. Linear regression fails to capture this difference because it averages these effects. Although it is easy to fit multiple QR models, it is important to assess the differences in QR coefficients statistically. The regression coefficients showed positive or negative trends in most cases, even though some of them were not statistically significant individually. This differential effect highlights the use of QR, and their significance is testable. However, significance testing in QR mostly relies on computer resampling or asymptotic approximations resting on distributional assumptions. The difficulty in interpreting multiple effects compared to a single measure of effect, as in OLS regression, is unavoidable (5). However, commonly used goodness-of-fit measures, such as the pseudo R-square and testing between two QR models via the ANOVA approach, as shown in the results, can be useful in model building and variable selection.

Most multivariable models showed different sets of significant predictors in our analysis. Supplementing well-established conditional mean models with QR to estimate an entire family of conditional quantile functions offers a much more complete statistical analysis of the relationships. Multivariable QR models can help address the fact that the variables that affect the lower parts of the distribution may not necessarily affect individuals at higher quantiles (28). It is worthwhile to explicitly find the set of covariates that affect a specific percentile (low or high) of the distribution to adopt a more specific approach based on the model. For example, if different treatments are to be compared for their effects on an outcome of interest, it is possible to determine whether the treatment effect is dependent on the level of the outcome itself. Such understanding can also help develop better models for predicting treatment response, as discussed by Katja et al. (16), and aid in deciding specific treatment approaches.

As a strictly illustrative case study of specific uses of QR in Psychiatry research, neuropsychological test performance in OCD has been explored. The neuropsychological literature on OCD is characterized by notable inconsistencies (11, 12, 29) that have not been satisfactorily explained by several clinical and demographic moderators such as age, education, gender, symptom subtypes, and severity of illness. While the variable CFT IR is most consistently known to be impaired in OCD, several others have shown inconsistent findings across studies. Our analyses show that, although CFT IR looks homogenous, varying QR coefficients across quantiles may help generate hypotheses about why CFT_IR appears consistently implicated across several studies. While the diagnosis of OCD itself may be linked to lower scores on the CFT IR compared to healthy controls (30), the impact of the severity of OCD (YBOCS score) seems to be most prominent at the lowest quantiles, possibly explaining why this finding seems to be the most consistently replicated across studies. In contrast, DST is not often reduced in OCD patients compared to controls (12). However, the QR suggests that the severity of OCD impacts attention in individuals performing at the highest quantiles. While attention may be impacted in subtle ways for most individuals with OCD, the impact of OCD severity may be most pronounced in high performers. The variables like DST, CTT2, and WCST_PR show an increasing absolute effect of age along the higher quantiles. The effect of age on WCST_TTCC1 appears in variations only in the middle quantiles. Despite symptomatic recovery, many individuals with OCD continue to experience cognitive deficits and accompanying socio-occupational dysfunction, necessitating targeted and personalized interventions for cognition. Despite some attempts at cognitive training in OCD, there has been limited success in generalizing improvements to socio-occupational functions (31). Analyses such as the QR presented here could potentially help explore the inconsistent findings, generate future hypotheses, and/or provide possible interpretive frameworks for such inconsistencies. A fine-grained, adequately powered analysis of neuropsychological findings using QR may generate a conceptual framework that provides richer insights that may help further research in this area. Furthermore, QR analyses may be better suited for the examination of individual differences in behavioral research and may make a meaningful contribution to the advancement of neuropsychological theory and the development of cognitive remediation/training interventions.

QR is a practical tool for analyzing complex relationships between variables because it extends beyond the scope of the central part of the distribution. While QR provides a complete picture of functional relationships, using QR to explore beyond the mean effects presents many new possibilities for statistical analysis and interpretation of relationships. There is an increasing trend in the use of QR in various fields of research, as can be seen by a simple PubMed search, and using QR for analyzing behavioral data can add to the existing knowledge substantially. Although it has begun to be used in many scientific applications, many researchers remain unaware of it because it was developed relatively recently and is rarely taught in statistics courses (32). The popularity of a statistical method is also dependent on its availability in software packages, and only a handful of software packages, such as R, SAS, and Stata, provide options to fit QR models. Advancements in computation techniques and the development of more user-friendly packages in open-source software, such as R, have made it easy to implement these methods.

## 5 Limitations

Several limitations should be considered when interpreting the findings of this study, considering its illustrative and exploratory nature. The use of a cross-sectional design and restricted geographic scope limits the ability to infer any causal relationships and the generalizability of the findings to broader or more diverse populations. Caution should be exercised when interpreting the effects of variables across quantiles, as cross-sectional studies typically reveal only conditional associations and not causal effects. The modest sample size (n = 119) may limit the statistical power to detect differences across quantiles, along with the stability of the estimates and generalizability of the findings. Furthermore, multiple testing corrections were not applied in this study, because of its exploratory objective. However, such corrections, such as simultaneous quantile regression (QR) or methods based on family wise error rate (FWER) or false discovery rate (FDR), should be considered when the goal is to identify the best-fitting model. Despite these limitations, the study provides an illustrative demonstration of QR applied to real clinical data and may help guide future methodological and empirical work, particularly longitudinal studies or those including broader and more diverse samples with comprehensive control of confounding factors and an appropriate sample size.

## Data Availability

The data analyzed in this study is a secondary dataset from a previous study at the National Institute of Mental Health and Neuro Sciences (NIMHANS), Bengaluru, India and it was used with necessary institutional permissions. The dataset is not readily available because of the sensitive nature of the information provided by the participants. Requests to access these datasets should be directed to Dr. Ravi GS at ravigs1988@gmail.com.
