# Supplementary material for: A case study on using quantile regression in psychiatry research
Source: Front Psychiatry. 2026 Jan 5;16:1632001. doi: 10.3389/fpsyt.2025.1632001 (PMC12812866; doi:10.3389/fpsyt.2025.1632001)
Supplement: Supplementary file 1 [file SupplementaryFile1.docx]

Supplementary Material

# Supplementary Materials

## Participant inclusion and exclusion criteria

Participants were included if they had a Yale-Brown Obsessive-Compulsive Scale (Y-BOCS) score ≥20, minimum illness duration of one year, seven or more years of education, and were right-handed and aged 18-45 years. Those with a history of tic disorder, psychosis, bipolar disorder, substance abuse, acquired brain injuries such as head injury, stroke, tumour or epilepsy, and clinical evidence of mental retardation were excluded.

Of the 150 participants analysed in the original study, 119 subjects with complete data were used for this analysis. Approximately two-thirds of the sample (66.4%) had at least one comorbid psychiatric disorder, most commonly depression (52.1%). At the time of assessment, approximately 80% of participants were receiving serotonin reuptake inhibitors (SRIs), mostly fluoxetine (29.9%), and 29.9% receiving Cognitive Behaviour Therapy. Almost half (44.5%) of the participants were receiving augmentation with an antipsychotic (14.3%) or a benzodiazepine (24.4%).

## Neuropsychological tests

The assessment included YBOCS and Clinical Global Impression (CGI) (Guy, 1976) for severity of OCD; the State Trait Anxiety Inventory (STAI) (Spielberger, 1983) Form Y for anxiety; the Hamilton Depression Rating Scale (HDRS-17) (Hamilton, 1960), for severity of depression.

A comprehensive battery of neuropsychological tests with established validity and reliability as listed below are administered.

**Attention:**

- Colour Trails Test (CTT) 1 and 2 for attention, visual scanning, and mental flexibility (Maj et. al., 1993)
- Digit Span for auditory attention and working memory (WMS III) (Wechsler, 1997a)
- Complex Figure Test (CFT) for nonverbal memory (Meyers and Meyers, 1995)

**Executive functions:**

- Tower of Hanoi Test (ToH) for planning (Welsh and Huizinga, 2005)
- Wisconsin Card Sorting Test (WCST) for concept formation and set shifting (Heaton et. al., 1993)

**References:**

- Maj, M., D'Elia, L., Satz, P., Janssen, R., Zaudig, M., Uchiyama, C., Starace, F., Galderisi, S., Chervinsky, A., & World Health Organization, Division of Mental Health/Global Programme on AIDS (1993). Evaluation of two new neuropsychological tests designed to minimize cultural bias in the assessment of HIV-1 seropositive persons: a WHO study. *Archives of clinical neuropsychology : the official journal of the National Academy of Neuropsychologists*, *8*(2), 123–135.
- Wechsler D. Wechsler Adult Intelligence Scale –Administration and scoring manual. 3. San Antonio, TX: 1997a.
- Meyers, J. E., & Meyers, K. R. (1995). Rey Complex Figure Test under four different administration procedures. *Clinical Neuropsychologist, 9*(1), 63–67.
- Marilyn C. Welsh, Mariëtte Huizinga, Tower of Hanoi disk-transfer task: Influences of strategy knowledge and learning on performance, Learning and Individual Differences, Volume 15, Issue 4, 2005, Pages 283-298, ISSN 1041-6080
- Heaton, R. K. (1993). Wisconsin card sorting test computer version 2.0. Odessa, FL: Psychological Assessment Resources.

# Supplementary Figures and Tables

## Supplementary Figures

***
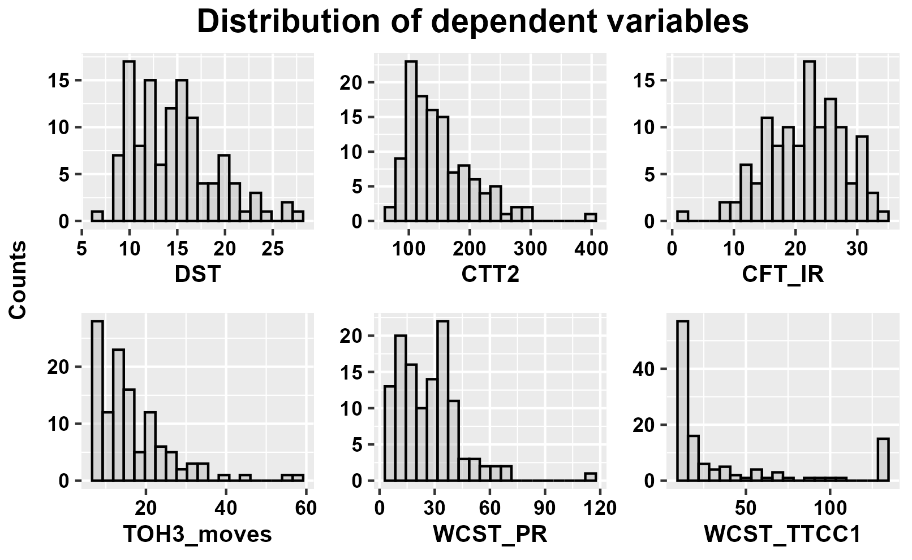
***

**Supplementary Figure 1: Distributions of test performances used as dependent variables in regression models**

**Supplementary Figure 2: Simple regression model with effect of age on test performances**

*
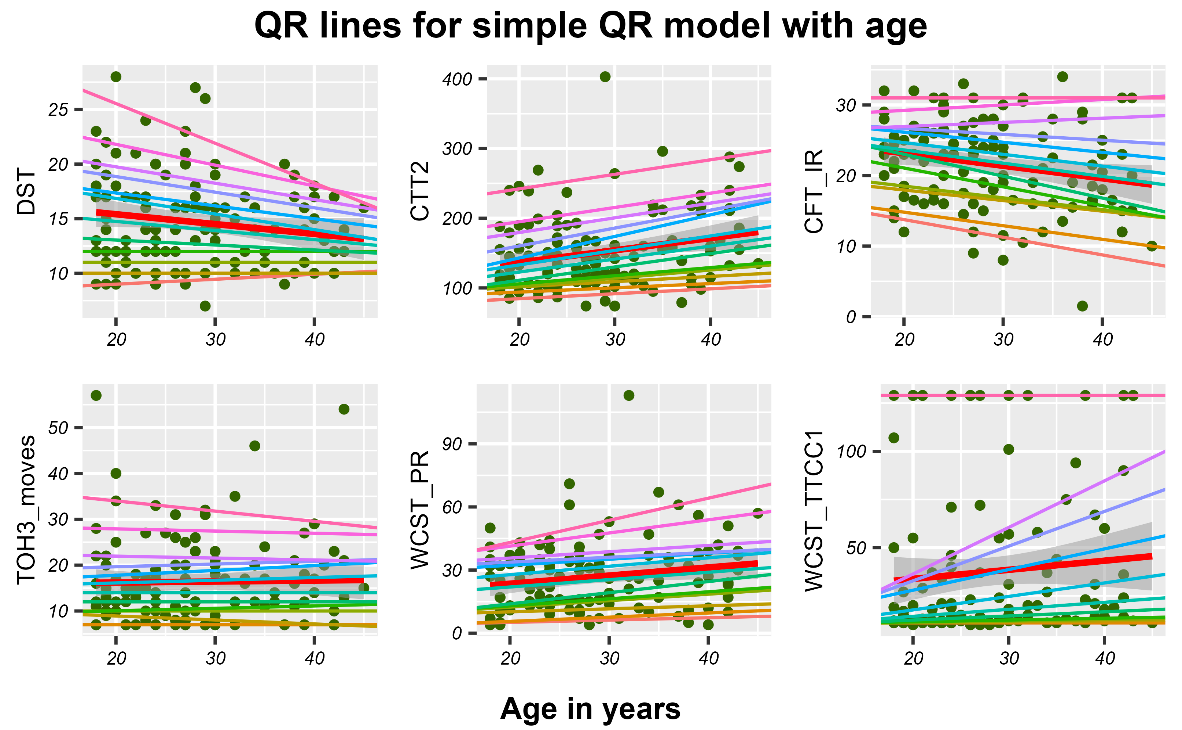
*

**Supplementary Figure 3: Comparison of change in regression coefficients across quantiles between a) Simple and b) Multivariable models**

**
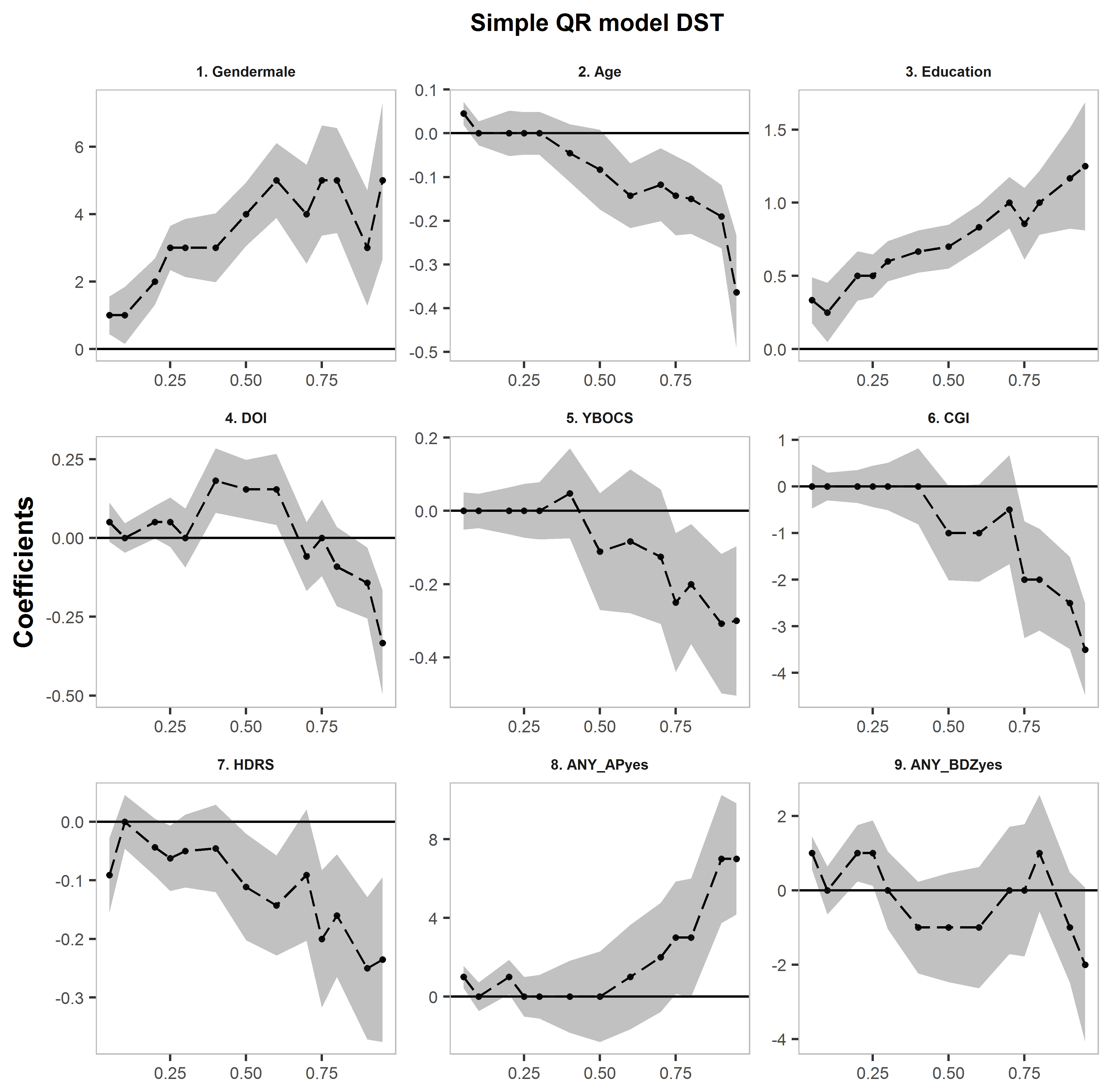

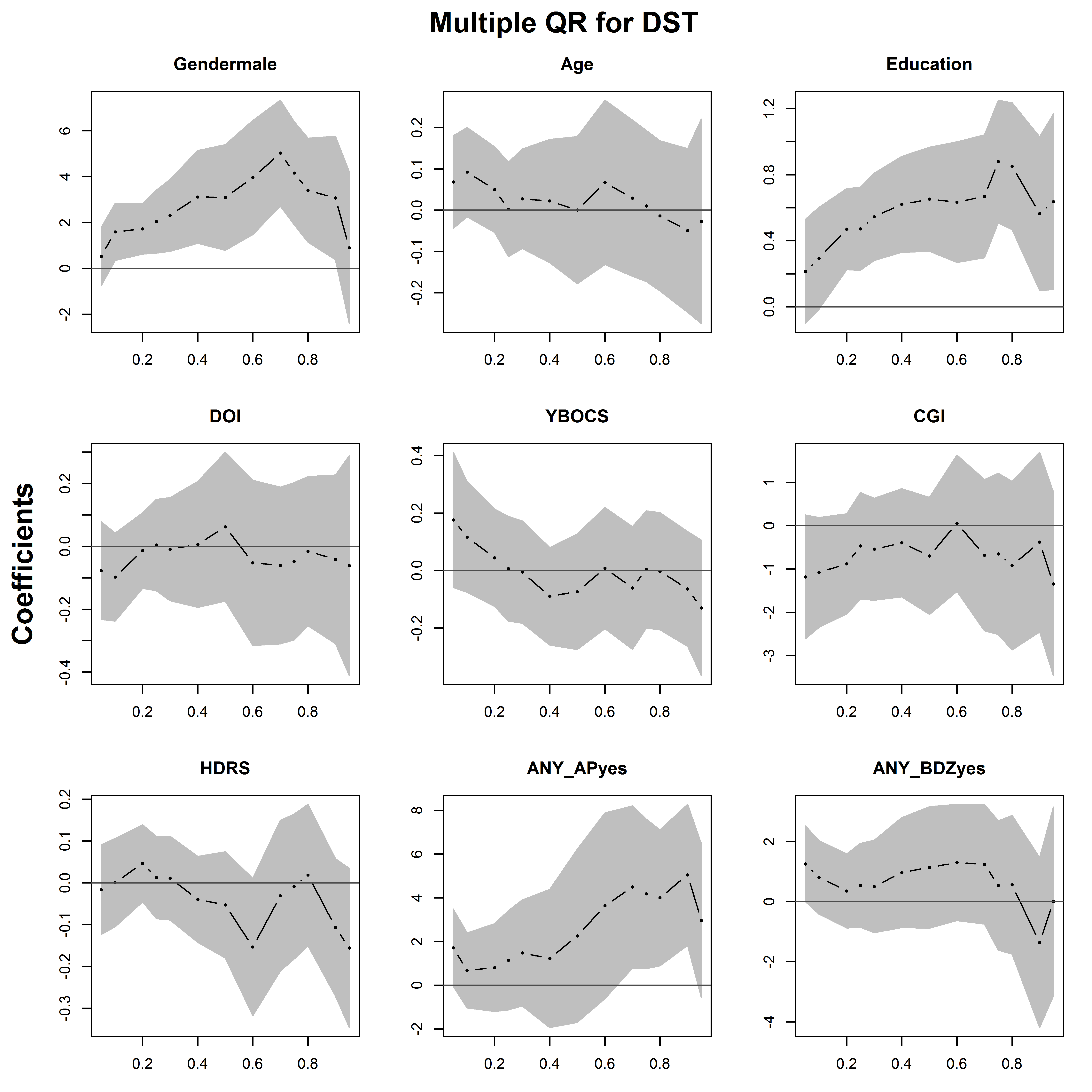
 *Comparison of unadjusted and adjusted QR coefficeints from Single and Multivariable OR Model – DST***

**
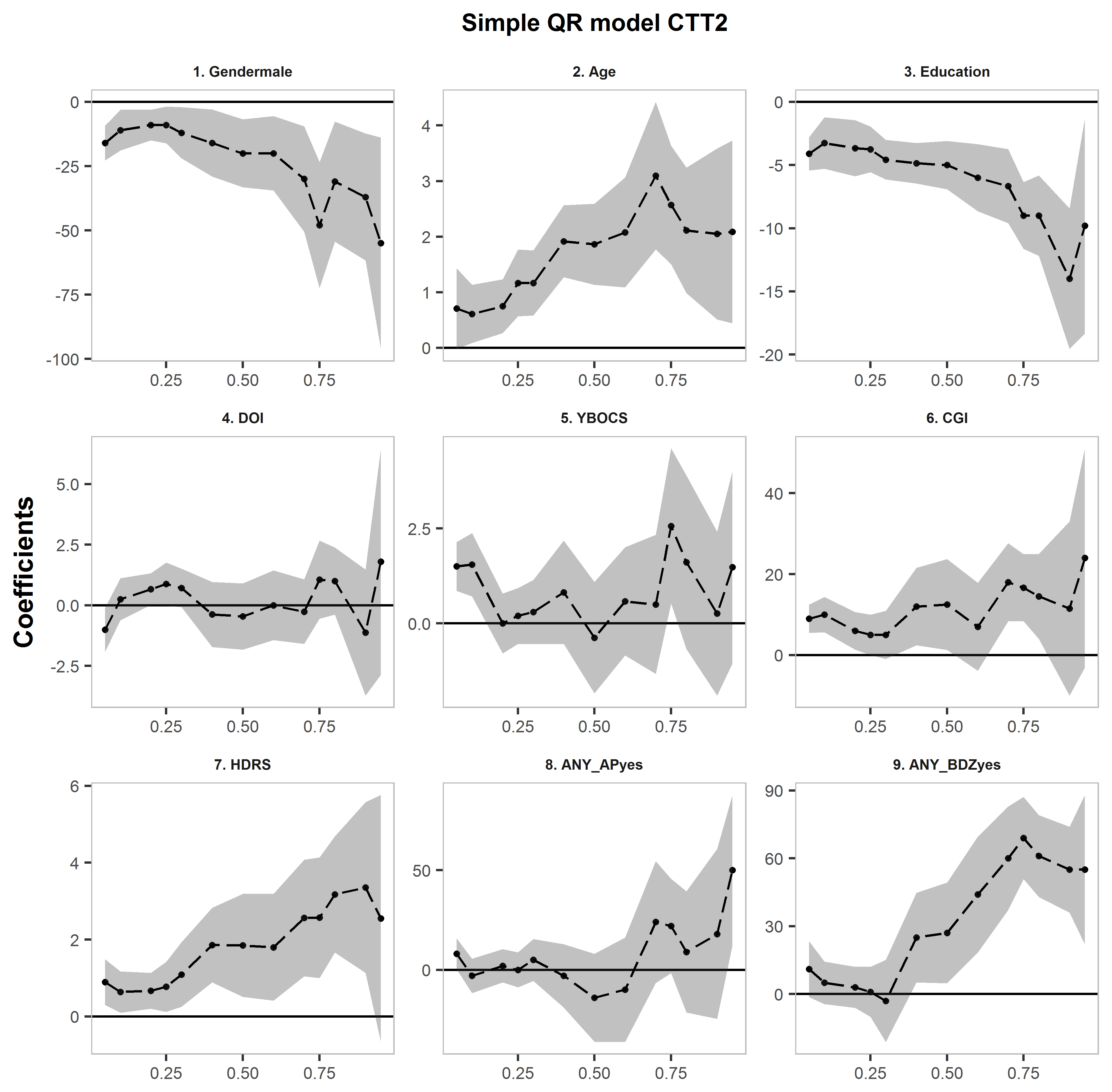

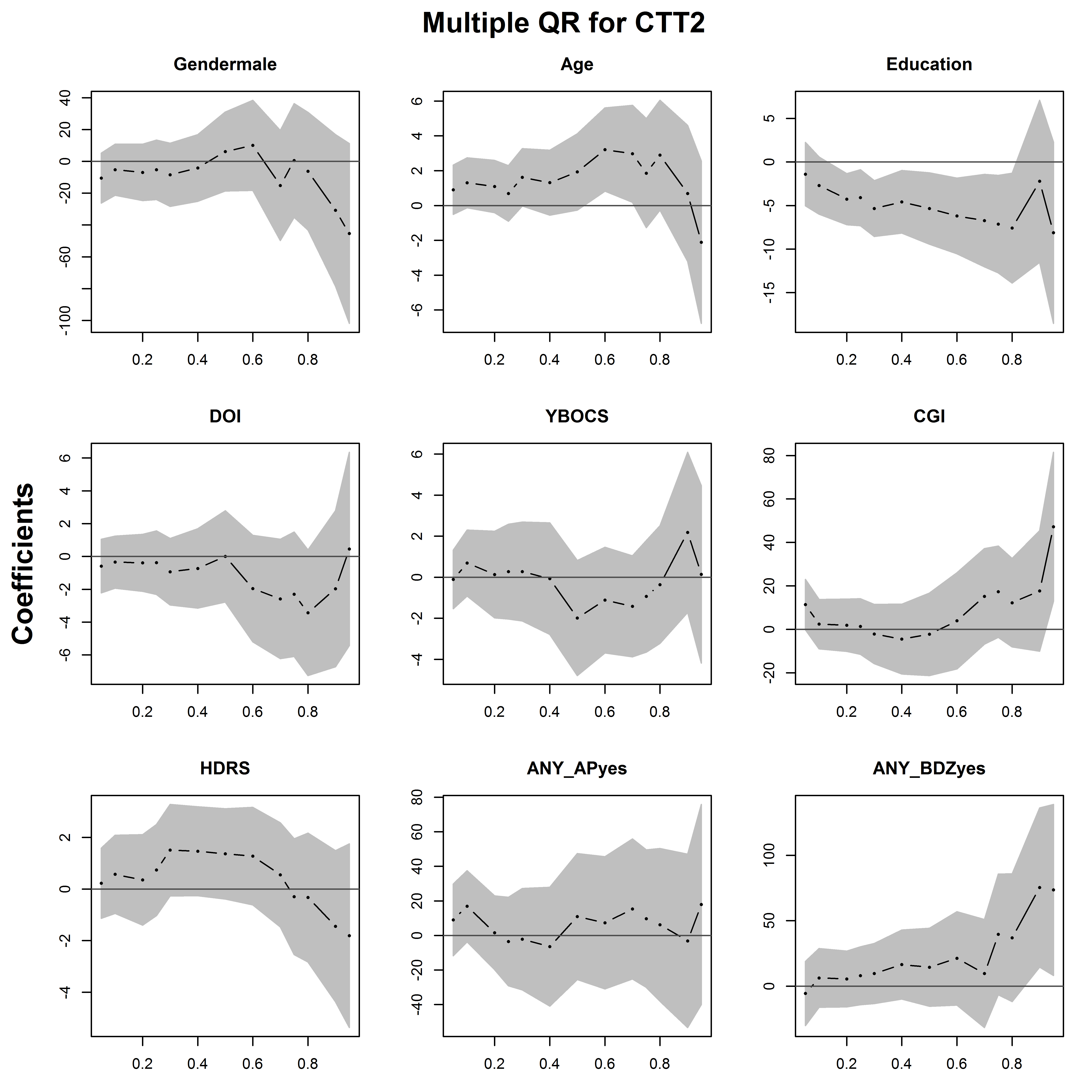
 *Comparison of unadjusted and adjusted QR coefficeints from Single and Multivariable OR Model – CTT2***

**
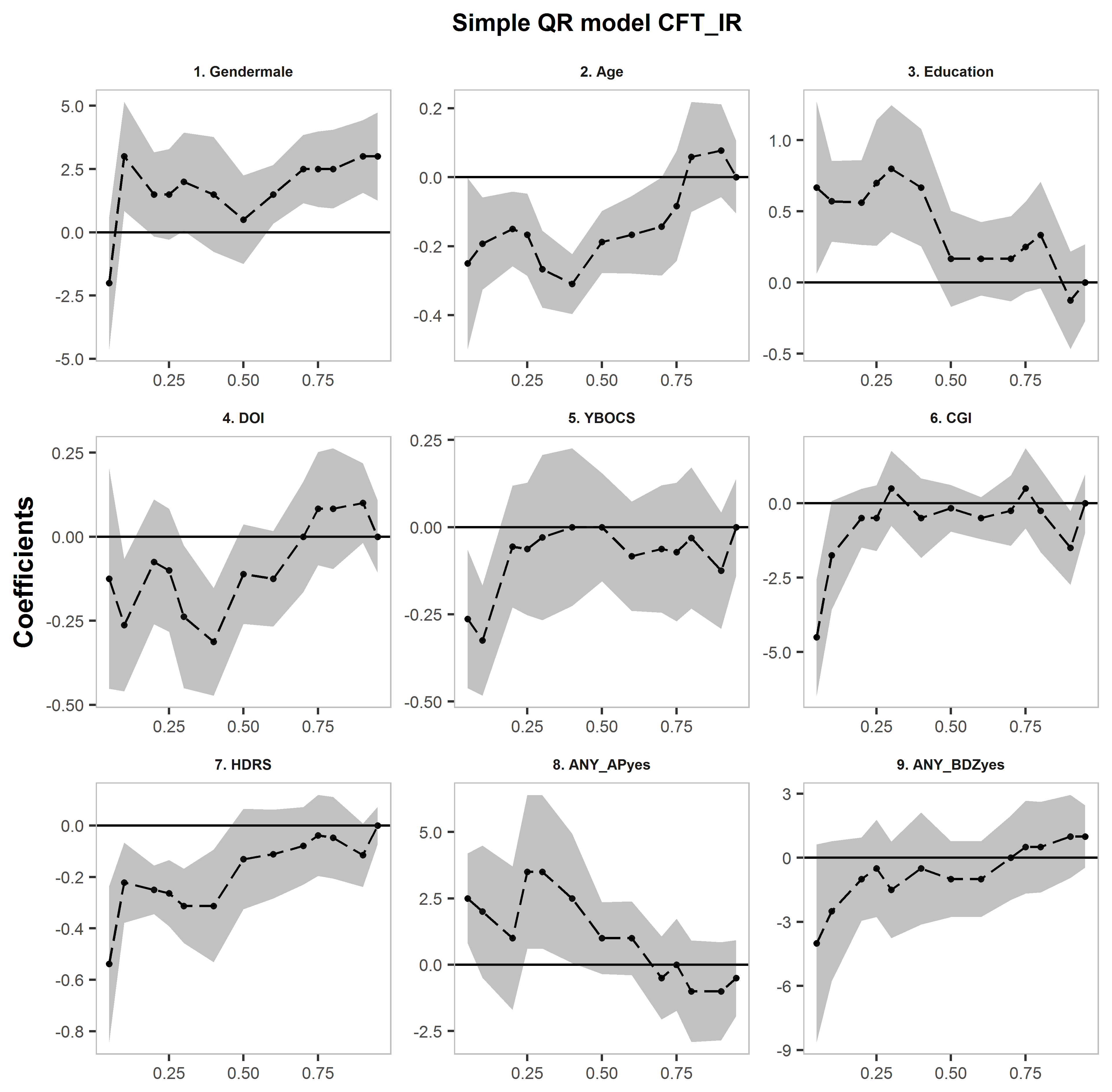

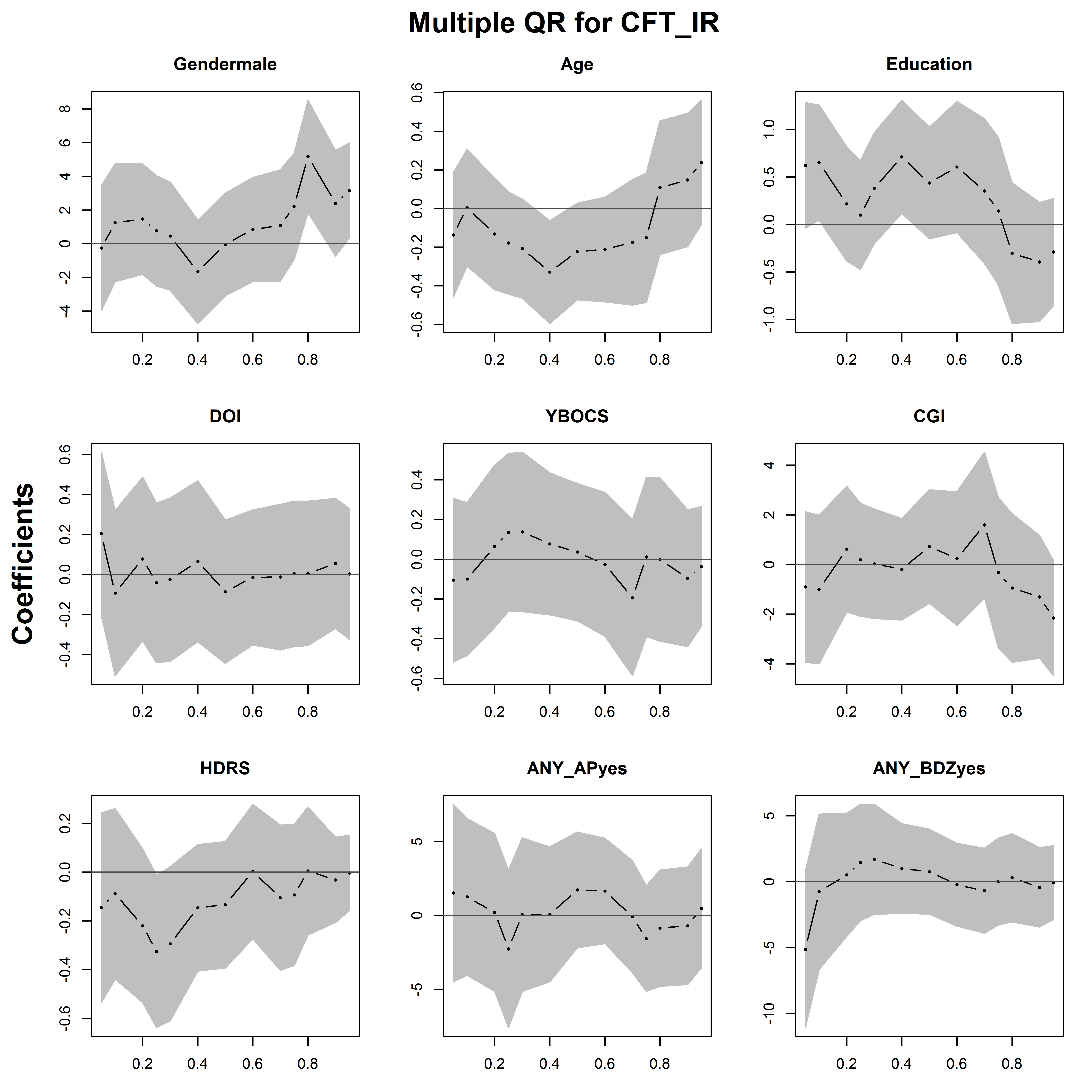
**

***Comparison of unadjusted and adjusted QR coefficeints from Single and Multivariable OR Model – CFT_IR***

**
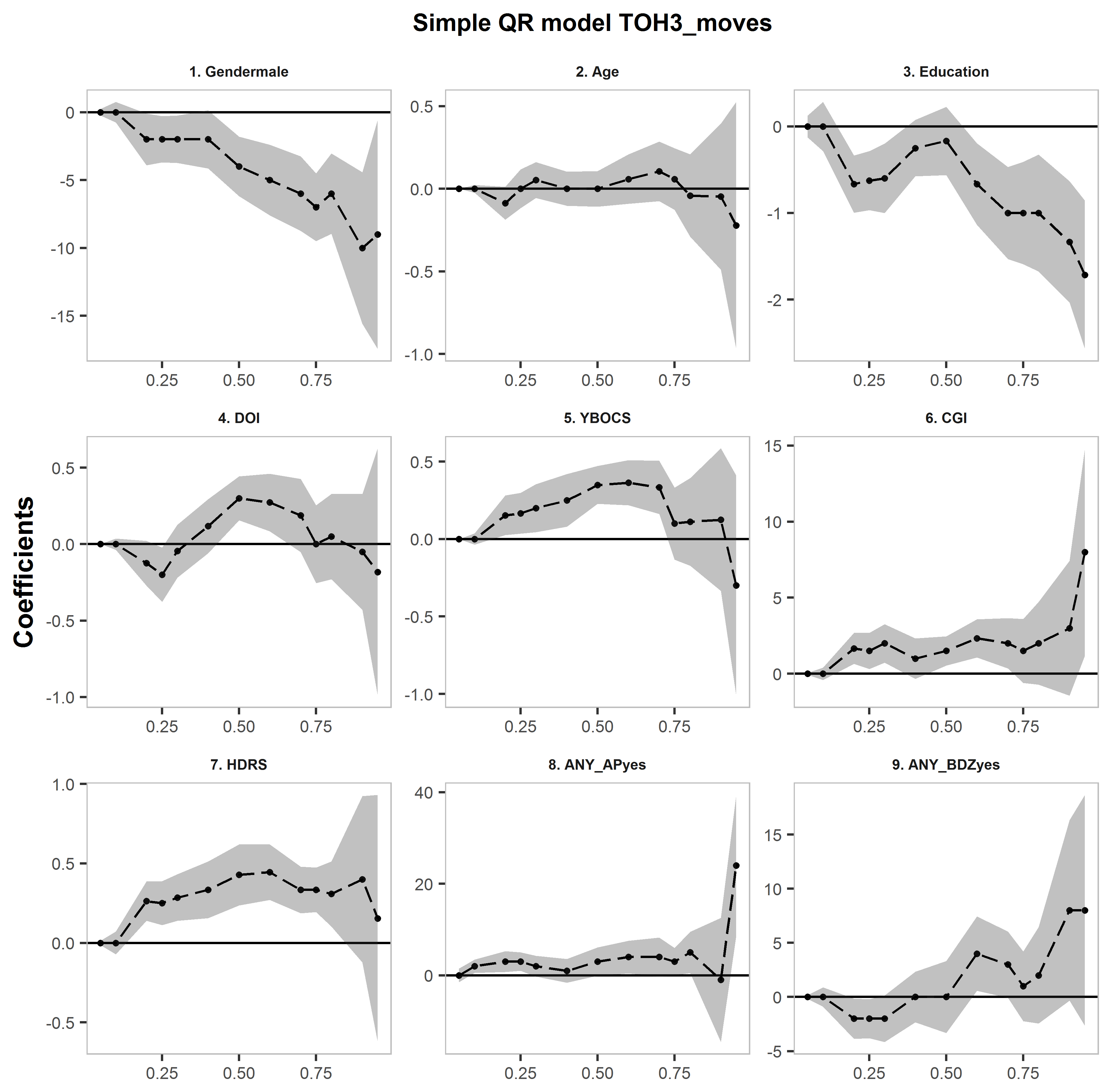

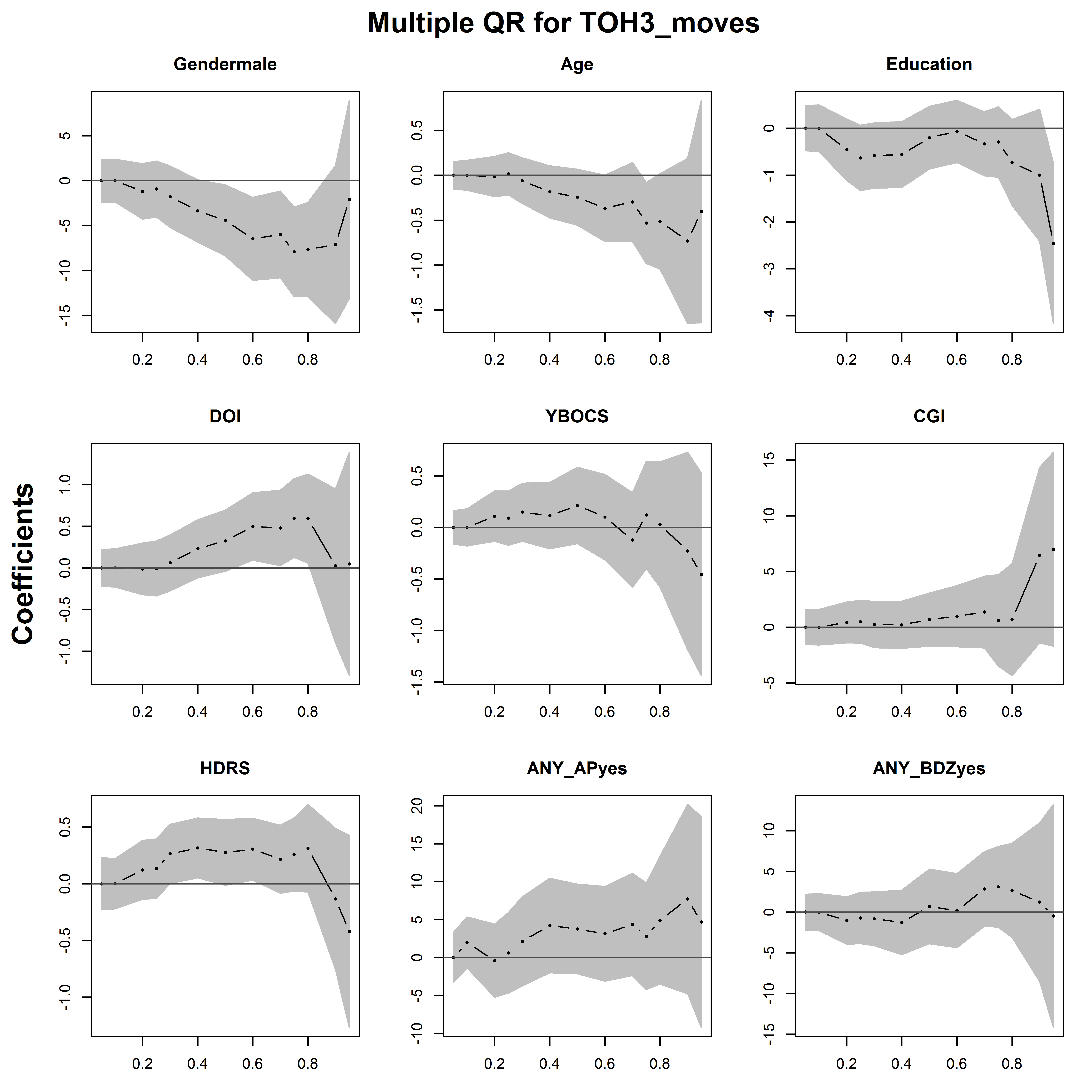
 *Comparison of unadjusted and adjusted QR coefficeints from Single and Multivariable OR Model – TOH3_moves***

**
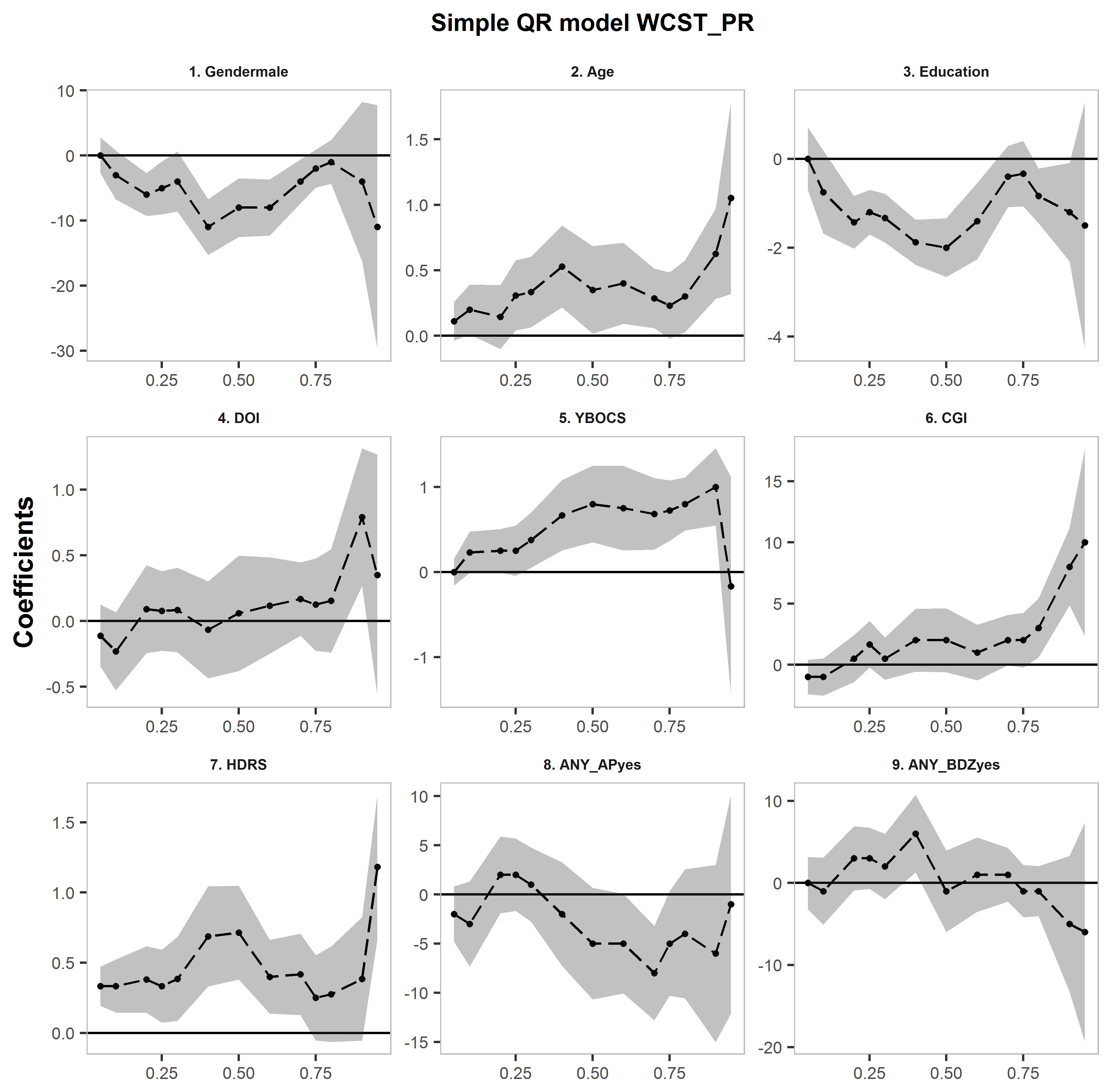

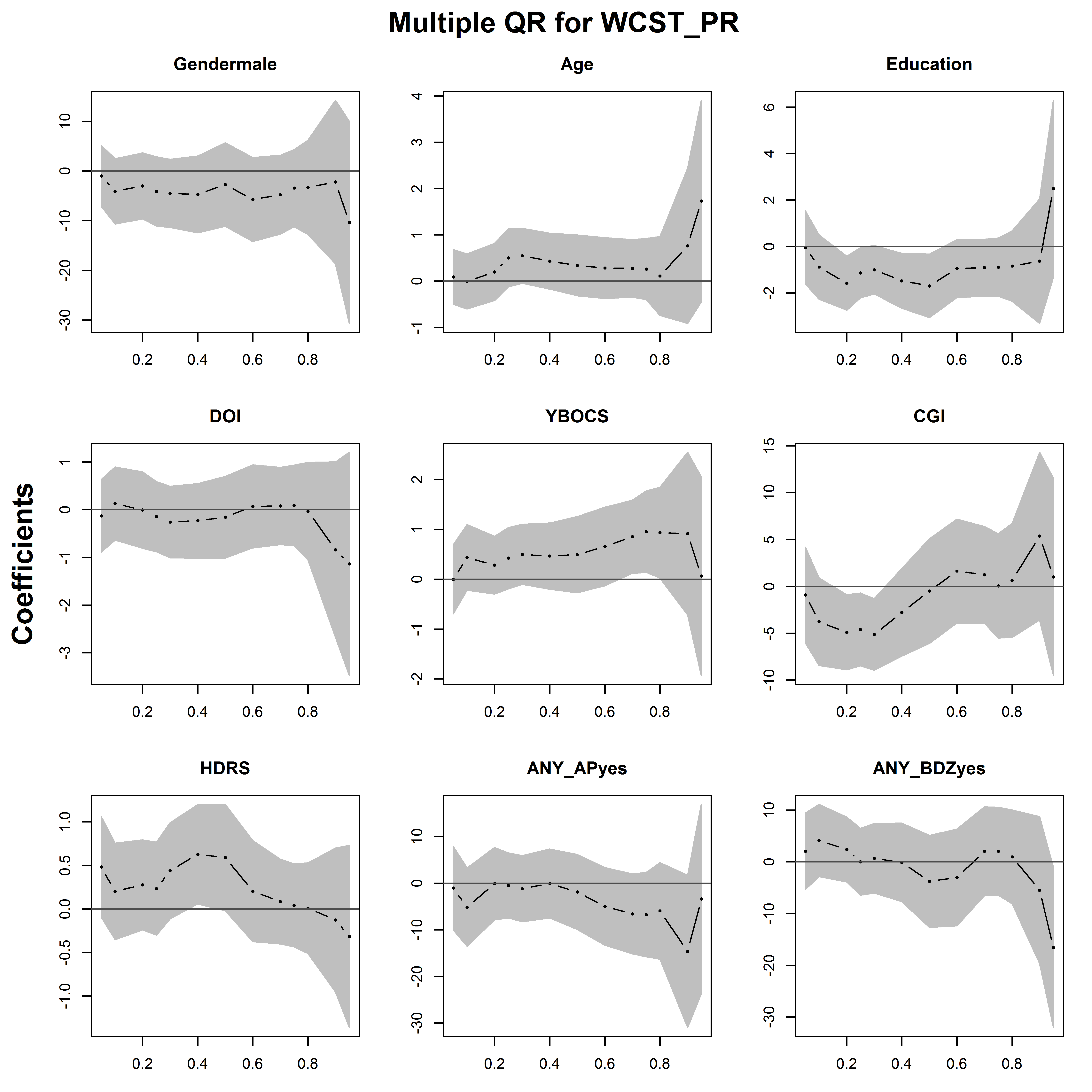
 *Comparison of unadjusted and adjusted QR coefficeints from Single and Multivariable OR Model – WCST_PR***

**
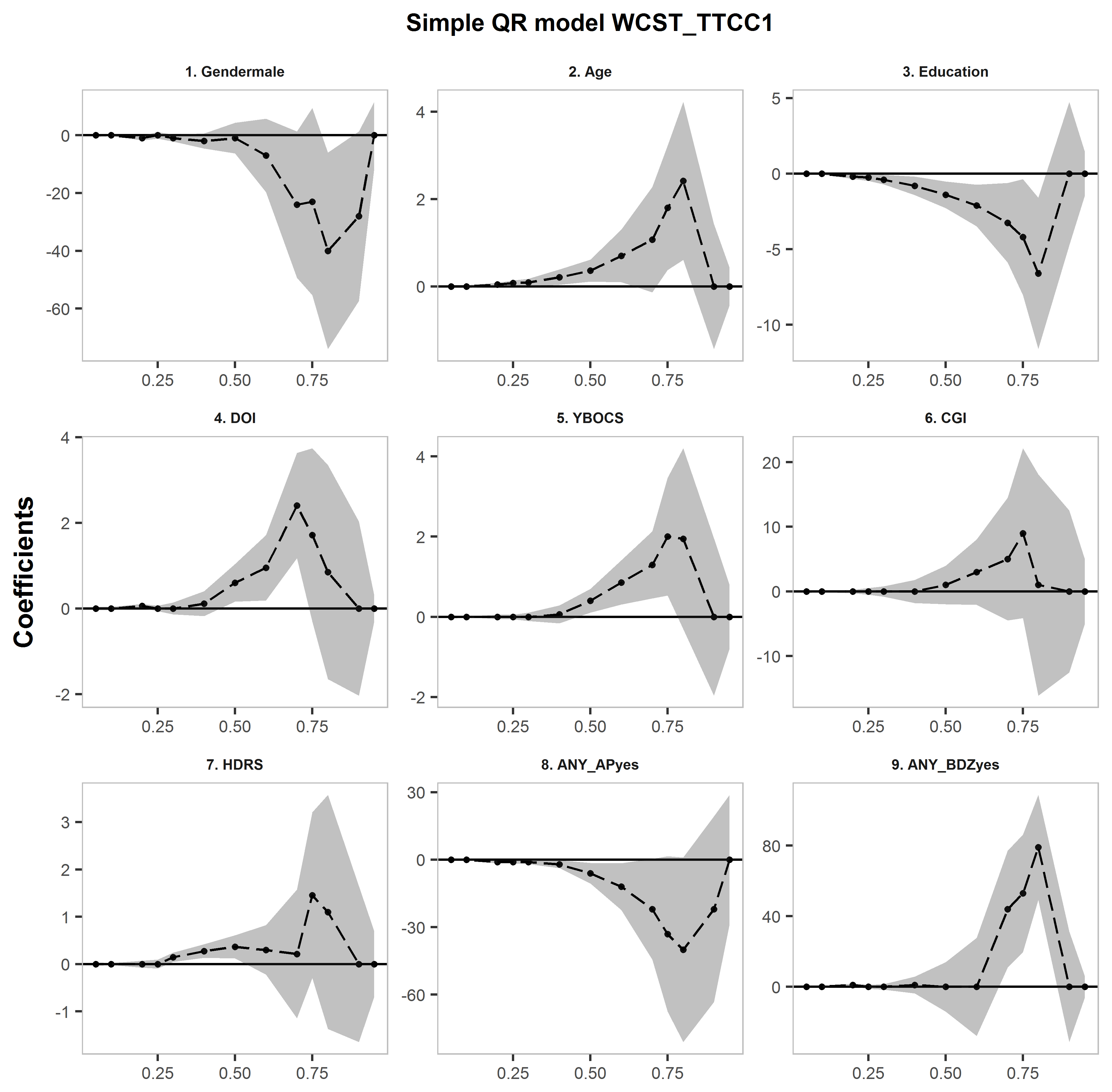

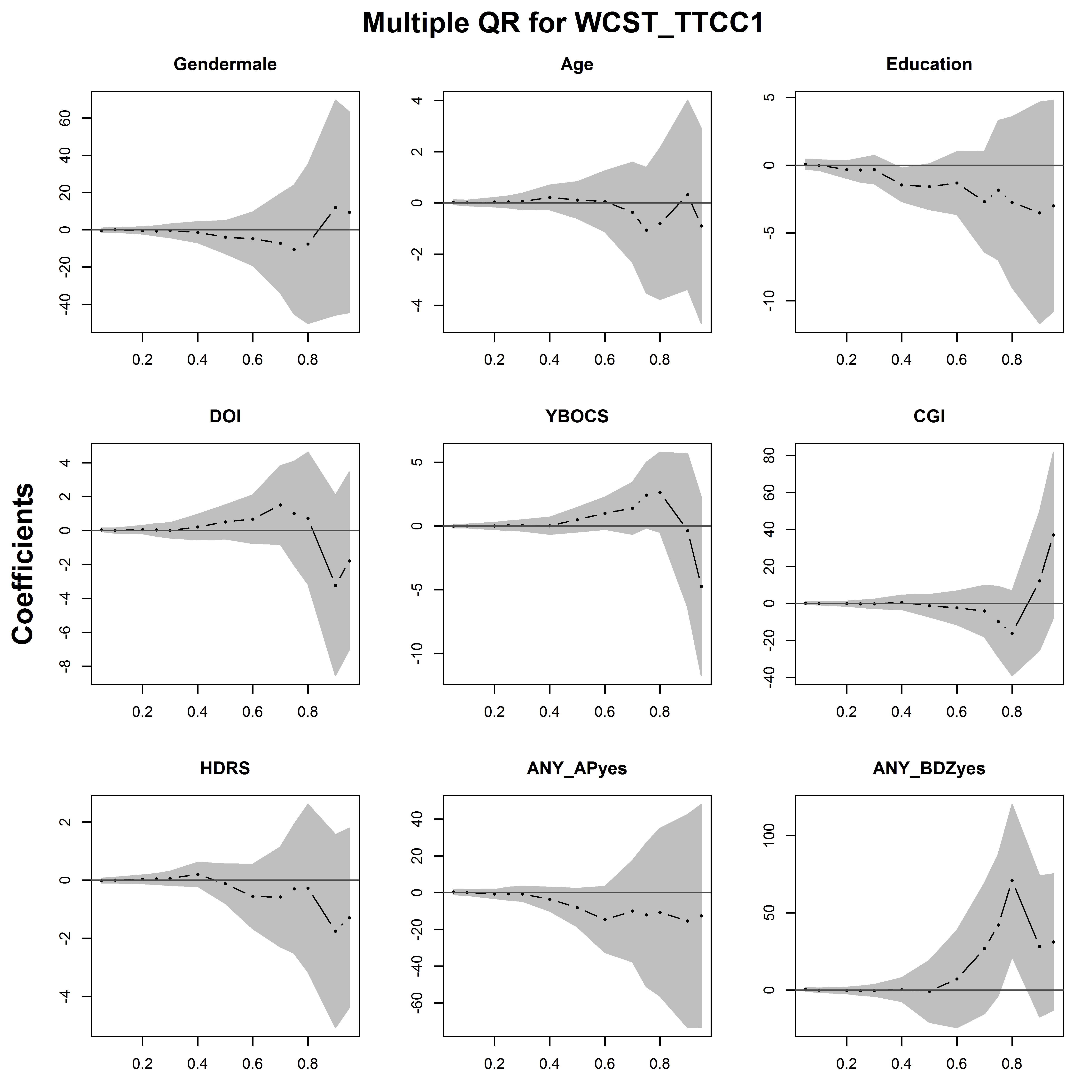
 *Comparison of unadjusted and adjusted QR coefficeints from Single and Multivariable OR Model – WCST_TTCC1***

**Supplementary Figure 4: Model fit indices for multivariable models**

## Supplementary Tables

**Supplementary Table 1: Socio-demographic and clinical characteristics of the study subjects (n=119)**

| ***Variable*** | ***Categories*** | ***Number (%)*** |
| --- | --- | --- |
| *Gender* | *Male* | *73 (61.3)* |
| *Insight* | *Good insight* | *82 (62.9)* |
| *Use of Antipsychotics* | *Yes* | *17 (14.3)* |
| *Use of Benzodiazepine* | *Yes* | *29 (24.4)* |
|  | ***Mean ± SD*** | ***Median: (Min-Max)*** |
| *Age (years)* | *28.24 ± 7.56* | *27: (18 - 45)* |
| *Duration of illness (years) (DOI)* | *8.55 ± 5.75* | *8: (1 - 24)* |
| *Age at onset (years)* | *19.71 ± 5.98* | *18: (10 - 36)* |
| *Education (years)* | *13.70 ± 2.60* | *14: (7-19)* |
| *CGIS* | *4.97 ± 0.78* | *5: (4 - 7)* |
| *HDRS* | *12.59 ± 6.43* | *12: (1 - 32)* |
| *YBOCS Total* | *27.76 ± 5.27* | *28: (11 - 40)* |
| *YBOCS-Obsession* | *14.01 ± 2.6* | *14: (8 - 20)* |
| *YBOCS-Compulsion* | *13.76 ± 3.62* | *14: (0 - 20)* |

*CGIS- Clinical Global Impression score, HDRS-Hamilton Depression Rating Scale, YBOCS-Yale Brown Obsessive Compulsive Scale*

**Supplementary Table 2: Descriptive statistics of test performances used as dependent variables in regression models**

| ***Variable*** | ***Mean ± SD*** | ***Median: (Range)*** | ***Quartiles*** | ***Test of Normality  (Shapiro-Wilk Test)*** | ***P-value for the test of residuals*** |
| --- | --- | --- | --- | --- | --- |
| *DST* | *14.66 ± 4.37* | *14: (7 - 28)* | *11; 14; 17* | *<0.001* | *0.001* |
| *CTT2* | *150.87 ± 55.2* | *141: (74 - 403)* | *109; 141; 179* | *<0.001* | *<0.001* |
| *CFT_IR* | *21.69 ± 6.27* | *23: (1.5 - 34)* | *16.5; 23; 26* | *0.101* | *0.05* |
| *TOH3_moves* | *16.39 ± 9.45* | *14: (7 - 57)* | *10; 14; 20* | *<0.001* | *<0.001* |
| *WCST_PR* | *26.69 ± 16.97* | *26: (4 - 113)* | *13; 26; 37* | *<0.001* | *<0.001* |
| *WCST_TTCC1* | *37.69 ± 40.24* | *18: (10 - 129)* | *12; 18; 46* | *<0.001* | *<0.001* |

*CTT2-Color Trails Test 2, DST-Digit Span Test, CFT-IR-Complex Figure Test Immediate Recall, TOH 3 moves-Tower of Hanoi Test number of moves for 3-disk problem, WCST-PR-Wisconsin Card Sorting Test -Perseverative errors, WCST-TTCC1-Wisconsin Card Sorting Test - trials taken to complete category 1*

**Supplementary Table 3: Multivariable QR model - Comparison of OLS and QR across selected quantiles for the multivariable QR model**

| **Dependent**  **Variable** | **Parameter** | ***OLS*** | ***Quantile values*** | | | | |  |
| --- | --- | --- | --- | --- | --- | --- | --- | --- |
|  |  |  | ***0.05*** | ***0.25*** | ***0.5*** | ***0.75*** | ***0.95*** |  |
| **DST** | **Intercept** | 7.86 (0.02) | 5.77 (0.050) | 5.78 (0.020) | 8.18 (0.060) | 5.57 (0.420) | 24.53 (0.030) |  |
|  | **Gender** | 2.75 (<0.001) | 0.52 (0.500) | 2.04 (0.010) | 3.09 (0.030) | 4.16 (<0.001) | 0.90 (0.650) |  |
|  | **Age** | -0.02 (0.740) | 0.07 (0.310) | 0.00 (0.980) | 0.00 (1.000) | 0.01 (0.930) | -0.03 (0.860) |  |
|  | **Education** | 0.67 (<0.001) | 0.21 (0.240) | 0.47 (<0.001) | 0.65 (<0.001) | 0.88 (<0.001) | 0.64 (0.060) |  |
|  | **DOI** | 0.02 (0.790) | -0.08 (0.420) | 0.00 (0.960) | 0.06 (0.670) | -0.05 (0.750) | -0.06 (0.780) |  |
|  | **YBOCS** | -0.02 (0.780) | 0.18 (0.190) | 0.01 (0.950) | -0.07 (0.540) | 0.00 (0.980) | -0.13 (0.350) |  |
|  | **CGI** | -0.64 (0.240) | -1.18 (0.140) | -0.47 (0.520) | -0.70 (0.400) | -0.65 (0.570) | -1.35 (0.290) |  |
|  | **HDRS** | -0.03 (0.620) | -0.02 (0.810) | 0.01 (0.840) | -0.05 (0.500) | -0.01 (0.930) | -0.16 (0.190) |  |
|  | **ANY_AP** | 2.49 (0.020) | 1.72 (0.110) | 1.14 (0.400) | 2.26 (0.340) | 4.18 (0.040) | 2.96 (0.180) |  |
|  | **ANY_BDZ** | 0.96 (0.240) | 1.26 (0.100) | 0.54 (0.510) | 1.14 (0.340) | 0.54 (0.660) | 0.01 (1.000) |  |
| **CTT2** | **Intercept** | 144.59 (<0.001) | 41.37 (0.460) | 132.07 (0.010) | 202.73 (<0.001) | 180.71 (0.020) | 199.85 (0.120) |  |
|  | **Gender** | -11.17 (0.290) | -10.53 (0.270) | -5.31 (0.640) | 6.19 (0.670) | 0.56 (0.980) | -45.35 (0.180) |  |
|  | **Age** | 1.68 (0.060) | 0.91 (0.280) | 0.69 (0.490) | 1.93 (0.150) | 1.86 (0.340) | -2.11 (0.460) |  |
|  | **Education** | -5.24 (0.010) | -1.39 (0.520) | -4.10 (0.030) | -5.35 (0.020) | -7.14 (0.050) | -8.13 (0.200) |  |
|  | **DOI** | -1.00 (0.370) | -0.58 (0.520) | -0.37 (0.750) | 0.00 (1.000) | -2.30 (0.340) | 0.46 (0.900) |  |
|  | **YBOCS** | -0.21 (0.850) | -0.11 (0.900) | 0.27 (0.850) | -1.99 (0.250) | -0.94 (0.540) | 0.14 (0.960) |  |
|  | **CGI** | 7.10 (0.350) | 11.37 (0.110) | 1.30 (0.870) | -2.30 (0.840) | 17.31 (0.190) | 47.20 (0.030) |  |
|  | **HDRS** | 0.93 (0.250) | 0.22 (0.790) | 0.73 (0.500) | 1.36 (0.180) | -0.30 (0.830) | -1.81 (0.400) |  |
|  | **ANY_AP** | 5.67 (0.690) | 9.05 (0.440) | -3.55 (0.820) | 10.94 (0.620) | 9.73 (0.700) | 17.94 (0.620) |  |
|  | **ANY_BDZ** | 16.86 (0.140) | -5.53 (0.700) | 8.02 (0.550) | 14.50 (0.440) | 39.62 (0.150) | 73.55 (0.060) |  |
| *Reference Category: Any AP and Any BDZ - No* | | | | | | | | |

| **Dependent**  **Variable** | **Parameter** | ***OLS*** | ***Quantile values*** | | | | |  |
| --- | --- | --- | --- | --- | --- | --- | --- | --- |
|  |  |  | ***0.05*** | ***0.25*** | ***0.5*** | ***0.75*** | ***0.95*** |  |
| **CFT_IR** | **Intercept** | 23.13 (<0.001) | 15.80 (0.140) | 20.52 (0.020) | 19.43 (0.010) | 29.76 (<0.001) | 37.40 (<0.001) |  |
|  | **Gender** | 0.68 (0.600) | -0.27 (0.910) | 0.76 (0.710) | -0.05 (0.980) | 2.21 (0.280) | 3.16 (0.090) |  |
|  | **Age** | -0.16 (0.140) | -0.14 (0.500) | -0.18 (0.280) | -0.22 (0.170) | -0.15 (0.460) | 0.24 (0.240) |  |
|  | **Education** | 0.31 (0.180) | 0.62 (0.140) | 0.10 (0.780) | 0.44 (0.200) | 0.14 (0.760) | -0.29 (0.410) |  |
|  | **DOI** | 0.02 (0.860) | 0.20 (0.420) | -0.04 (0.860) | -0.09 (0.680) | 0.00 (0.990) | 0.00 (0.990) |  |
|  | **YBOCS** | -0.04 (0.770) | -0.11 (0.690) | 0.14 (0.590) | 0.04 (0.870) | 0.01 (0.960) | -0.04 (0.850) |  |
|  | **CGI** | 0.09 (0.920) | -0.90 (0.620) | 0.20 (0.890) | 0.72 (0.610) | -0.32 (0.860) | -2.15 (0.140) |  |
|  | **HDRS** | -0.10 (0.320) | -0.15 (0.550) | -0.33 (0.080) | -0.13 (0.390) | -0.09 (0.600) | 0.00 (0.980) |  |
|  | **ANY_AP** | 0.40 (0.82) | 1.52 (0.680) | -2.27 (0.460) | 1.73 (0.490) | -1.57 (0.500) | 0.47 (0.850) |  |
|  | **ANY_BDZ** | -0.12 (0.93) | -5.13 (0.140) | 1.46 (0.580) | 0.77 (0.680) | 0.01 (0.990) | -0.06 (0.970) |  |
| ***TOH3_moves*** | **Intercept** | 18.41 (0.030) | 40.79 (0.510) | 42.83 (0.610) | 60.69 (0.550) | 177.00 (0.250) | -179.56 (0.560) |  |
|  | **Gender** | -4.26 (0.030) | -5.24 (0.660) | -23.45 (0.180) | -61.97 (0.010) | -89.73 (0.020) | -98.73 (0.050) |  |
|  | **Age** | -0.17 (0.280) | 0.32 (0.720) | -0.02 (0.990) | -1.38 (0.410) | -1.97 (0.580) | 11.93 (0.160) |  |
|  | **Education** | -0.51 (0.140) | -2.17 (0.370) | -2.51 (0.490) | -2.09 (0.590) | -6.08 (0.280) | -8.80 (0.290) |  |
|  | **DOI** | 0.20 (0.320) | -0.36 (0.740) | 1.05 (0.540) | 2.63 (0.170) | 1.20 (0.750) | -5.85 (0.460) |  |
|  | **YBOCS** | 0.00 (0.990) | -0.81 (0.380) | 0.58 (0.640) | 0.44 (0.830) | 2.01 (0.520) | -0.94 (0.850) |  |
|  | **CGI** | 1.69 (0.220) | 4.31 (0.570) | -0.46 (0.970) | 9.76 (0.550) | 12.07 (0.620) | 64.90 (0.070) |  |
|  | **HDRS** | 0.14 (0.330) | 0.62 (0.570) | 2.89 (0.030) | 3.64 (0.020) | 2.33 (0.280) | 2.49 (0.700) |  |
|  | **ANY_AP** | 2.37 (0.360) | 23.23 (0.100) | 26.22 (0.280) | 34.68 (0.320) | 59.21 (0.220) | 78.37 (0.230) |  |
|  | **ANY_BDZ** | 0.37 (0.860) | 8.60 (0.460) | 7.00 (0.670) | 9.68 (0.630) | -4.46 (0.870) | 33.73 (0.760) |  |
| *Reference Category: Any AP and Any BDZ - No* | | | | | | | | |

| **Dependent**  **Variable** | **Parameter** | ***OLS*** | ***Quantile values*** | | | | |  |
| --- | --- | --- | --- | --- | --- | --- | --- | --- |
|  |  |  | ***0.05*** | ***0.25*** | ***0.5*** | ***0.75*** | ***0.95*** |  |
| ***WCST_PR*** | ***Intercept*** | 27.02 (0.070) | 4.53 (0.830) | 29.74 (0.070) | 23.37 (0.200) | 13.98 (0.430) | -8.10 (0.880) |  |
|  | ***Gender*** | -3.58 (0.300) | -1.00 (0.790) | -4.09 (0.320) | -2.73 (0.610) | -3.45 (0.490) | -10.35 (0.420) |  |
|  | ***Age*** | 0.41 (0.160) | 0.09 (0.790) | 0.50 (0.180) | 0.34 (0.420) | 0.26 (0.510) | 1.73 (0.190) |  |
|  | ***Education*** | -1.09 (0.080) | -0.04 (0.970) | -1.13 (0.090) | -1.70 (0.040) | -0.90 (0.250) | 2.49 (0.270) |  |
|  | ***DOI*** | -0.27 (0.460) | -0.13 (0.780) | -0.15 (0.750) | -0.16 (0.760) | 0.09 (0.850) | -1.14 (0.410) |  |
|  | ***YBOCS*** | 0.40 (0.270) | 0.00 (0.990) | 0.42 (0.270) | 0.49 (0.280) | 0.95 (0.050) | 0.06 (0.960) |  |
|  | ***CGI*** | -1.24 (0.620) | -0.93 (0.770) | -4.60 (0.040) | -0.51 (0.880) | 0.05 (0.990) | 1.01 (0.870) |  |
|  | ***HDRS*** | 0.28 (0.280) | 0.48 (0.160) | 0.23 (0.450) | 0.59 (0.110) | 0.04 (0.890) | -0.32 (0.640) |  |
|  | ***ANY_AP*** | -2.87 (0.540) | -1.06 (0.840) | -0.50 (0.910) | -1.89 (0.710) | -6.74 (0.220) | -3.40 (0.790) |  |
|  | ***ANY_BDZ*** | -1.78 (0.630) | 2.07 (0.660) | 0.02 (1.000) | -3.77 (0.480) | 2.06 (0.680) | -16.59 (0.090) |  |
| ***WCST_TTCC1*** | ***Intercept*** | 60.42 (0.100) | 8.99 (0.030) | 16.46 (0.040) | 32.79 (0.180) | 76.24 (0.320) | 157.03 (0.200) |  |
|  | ***Gender*** | -8.8 (0.290) | -0.27 (0.710) | -0.60 (0.710) | -3.98 (0.470) | -10.61 (0.610) | 9.45 (0.770) |  |
|  | ***Age*** | -0.07 (0.920) | 0.03 (0.620) | 0.04 (0.790) | 0.11 (0.800) | -1.07 (0.450) | -0.90 (0.700) |  |
|  | ***Education*** | -1.94 (0.200) | 0.07 (0.740) | -0.36 (0.500) | -1.59 (0.130) | -1.84 (0.550) | -3.00 (0.540) |  |
|  | ***DOI*** | 0.33 (0.701) | 0.04 (0.500) | 0.04 (0.860) | 0.51 (0.400) | 1.02 (0.560) | -1.79 (0.590) |  |
|  | ***YBOCS*** | 1.04 (0.240) | -0.02 (0.770) | 0.03 (0.890) | 0.49 (0.390) | 2.41 (0.100) | -4.74 (0.270) |  |
|  | ***CGI*** | -3.36 (0.580) | 0.09 (0.830) | -0.27 (0.820) | -1.28 (0.730) | -9.86 (0.340) | 36.95 (0.170) |  |
|  | ***HDRS*** | -0.36 (0.570) | -0.02 (0.720) | 0.04 (0.760) | -0.12 (0.760) | -0.30 (0.830) | -1.30 (0.480) |  |
|  | ***ANY_AP*** | -14.22 (0.201) | 0.33 (0.710) | -0.55 (0.780) | -8.10 (0.180) | -12.03 (0.590) | -12.57 (0.730) |  |
|  | ***ANY_BDZ*** | 12.31 (0.170) | 0.47 (0.530) | -0.31 (0.870) | -0.73 (0.950) | 42.19 (0.130) | 31.22 (0.240) |  |
| *Reference Category: Any AP and Any BDZ - No* | | | | | | | | |
